# Joint superpixel and Transformer for high resolution remote sensing image classification

**DOI:** 10.1038/s41598-024-55482-y

**Published:** 2024-03-01

**Authors:** Guangpu Dang, Zhongan Mao, Tingyu Zhang, Tao Liu, Tao Wang, Liangzhi Li, Yu Gao, Runqing Tian, Kun Wang, Ling Han

**Affiliations:** 1 Shaanxi Provincial Land Engineering Construction Group Land Survey Planning and Design Institute, Xi’an, Shaanxi China; 2 Shaanxi Provincial Land Engineering Construction Group, Xi’an, Shaanxi China; 3 Key Laboratory of Degraded and Unused Land Consolidation Engineering, the Ministry of Natural Resources, Xi’an, Shaanxi China; 4 Institute of Land Engineering and Technology, Shaanxi Provincial Land Engineering Construction Group, Xi’an, Shaanxi China; 5 Land Reserve Center of High tech Development Zone, Xi’an, Shaanxi China; 6 Chang’an University, Xi’an, Shaanxi China

**Keywords:** Remote sensing image, Image classification, Superpixel, Transformer, Deep learning, Environmental sciences, Space physics, Environmental sciences, Space physics

## Abstract

Deep neural networks combined with superpixel segmentation have proven to be superior to high-resolution remote sensing image (HRI) classification. Currently, most HRI classification methods that combine deep learning and superpixel segmentation use stacking on multiple scales to extract contextual information from segmented objects. However, this approach does not take into account the contextual dependencies between each segmented object. To solve this problem, a joint superpixel and Transformer (JST) framework is proposed for HRI classification. In JST, HRI is first segmented into superpixel objects as input, and Transformer is used to model the long-range dependencies. The contextual relationship between each input superpixel object is obtained and the class of analyzed objects is output by designing an encoding and decoding Transformer. Additionally, we explore the effect of semantic range on classification accuracy. JST is also tested by using two HRI datasets with overall classification accuracy, average accuracy and Kappa coefficients of 0.79, 0.70, 0.78 and 0.91, 0.85, 0.89, respectively. The effectiveness of the proposed method is compared qualitatively and quantitatively, and the results achieve competitive and consistently better than the benchmark comparison method.

## Introduction

The resolution of acquired remote sensing images is increasing as remote sensing sensors and imaging technology advance^1,2^. In comparison to the earlier low and medium resolution images, HRI can offer greater spatial information and geometric texture information^3,4^. It offers trustworthy information for land management, land planning, and urban construction^5^. Additionally, this raises the bar for high-resolution remote sensing image classification^6,7^.

**Figure 1:**
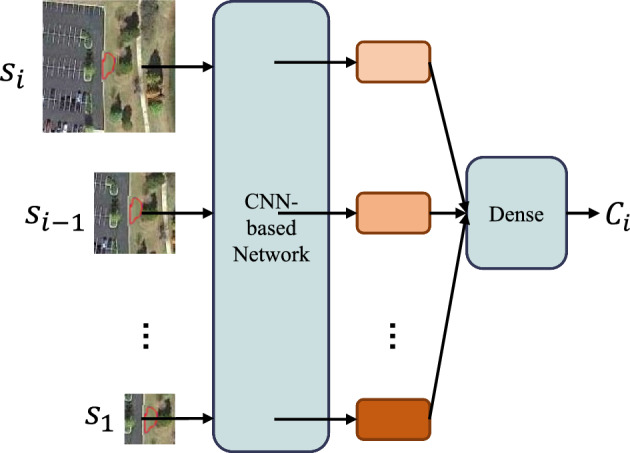
Illustration of a superpixel object for multi-scale input to deep neural networks.

Currently, research on deep learning-based methods has increased rapidly as deep neural networks have achieved significant advances^8–11^. Several frameworks have been developed in combination with deep learning-based methods, including autoencoders^12^, constrained Boltzmann machines^13^, and convolutional neural networks (CNNs)^14^. Specifically, CNNs are more widely used in remote sensing image classification.^15^ proposed a CNN framework for remote sensing image classification. The framework extracts deep features using a series of CNN and pooling layers to improve the accuracy of remote sensing image classification.^16^ used a pyramidal pooling module to enable CNNs to combine multi-scale information for remote sensing image classification. This method can recognize multiple geographical objects simultaneously. Researches mentioned above demonstrate how the deep learning-based remote sensing image classification method enhances accuracy and lessens issues with conventional feature extraction and feature selection.

Despite the aforesaid benefits of deep learning-based methods, HRI classification still has significant drawbacks. End-to-end semantic segmentation networks are mostly used in deep learning-based remote sensing image classification to achieve pixel-level classification^17–20^. For complicated feature objects, these semantic segmentation approaches have a pretzel effect since it is challenging to determine the correct class for each pixel^21,22^.

In contrast, the above scenario is avoided from the object level using the superpixel segmentation and deep neural network classification approach^23,24^.^25^ proposed a deep learning method based on CNN and energy-driven sampling for high-resolution remote sensing image classification.^26^ proposed a deep neural network method for standardized segmentation of objects for HRI classification. These superpixel-based classification methods can effectively map image classes with high spatial resolution. However, these methods only stack multiple scale images for the feature presentation, which not only increases the redundancy of information, but also increases the non-separability between features.

Figure 1 shows stacking multiple scales of image blocks for characterizing the central superpixel object features. $S_{1}$, $S_{2}$,..., $S_{i}$ scales are sampled and these data are simultaneously used as input as feature information for the classification of this superpixel block. This approach only obtains a class of superpixel objects, while not obtaining the remote dependence of a superpixel object.

Although some progress has been made in HRI classification based on superpixel segmentation, it is still worth exploring. The main problems are as follows:

1. *Semantic range.* HRI typically contain features at several scales, therefore using images with a set scale range as input will add to the complexity of representing heterogeneous information when the network is trying to extract features from objects at various scales.
2. *Context dependency.* It fails to determine the class to which the object belongs using only one superpixel object, and the context dependency between him and the surrounding objects must be captured.

To address the above problems, this paper proposes a framework of joint superpixel and Transformer^27^ is proposed for HRI classification. Transformer structure can reflect the complex spatial transformation and contextual dependency to obtain the global feature representation. Inspired by the above, we designed an encoded and decoded Transformer to obtain the contextual relationship between each input superpixel object and output the class of analyzed objects. The main contributions are as follows:

1. Semantic Scaling through Superpixel Object Selection: Our framework addresses the issue of semantic scaling by selecting different numbers of superpixel objects as inputs. This approach allows for the representation of scale differences between superpixel objects, a crucial factor in HRI classification. By adapting the scale of input superpixel objects, our method can more accurately and effectively capture the varying semantic levels present in HRIs.
2. Encoded and Decoded Transformer Design: We have innovatively designed a Transformer structure for encoding and decoding, which is inspired by the need to capture complex spatial transformations and contextual dependencies. This design enables the Transformer to obtain a global feature representation of the input data. By establishing contextual dependencies, our Transformer incrementally enhances the understanding of the relationships between objects and their surrounding context. This aspect is pivotal in accurately classifying each superpixel object based on a comprehensive understanding of its context.

## Related work

### Image classification techniques

Traditional image classification techniques can be broadly categorized into supervised and unsupervised methods^28^. Supervised classification methods, such as maximum likelihood classification (MLC) and support vector machine (SVM), rely on labeled training data to create a model that can predict the class labels of unseen data^29^. These methods have been widely used in remote sensing image classification tasks due to their high accuracy when training data is representative and adequately labeled. However, they require a large amount of labeled training data, which can be expensive and time-consuming to collect^30^. Moreover, these methods may not generalize well to new datasets or when the class distributions change over time.

Unsupervised classification methods, such as K-means Clustering and Iterative Self-Organizing Data Analysis Technique (ISODATA), do not require labeled data and are based on clustering algorithms to group pixels with similar characteristics^31^. These methods are advantageous when labeled data is scarce, but their performance heavily relies on the choice of the clustering algorithm and its parameters. As a result, unsupervised methods may produce less accurate classification results compared to supervised methods^32^.

Feature extraction techniques play a crucial role in remote sensing image classification, as they determine the representation of the data used for classification. Handcrafted features, such as texture features (e.g., Gray Level Co-occurrence Matrix, GLCM) and spectral features (e.g., vegetation indices), involve manual selection and extraction of features based on domain knowledge^33^. These features have been widely used in remote sensing image classification tasks due to their ability to capture relevant information, such as spatial patterns and spectral characteristics. However, the selection of appropriate features is a challenging task, and handcrafted features may not capture all the information required for accurate classification^34^.

Deep learning-based feature extraction methods, particularly Convolutional Neural Networks (CNNs), have revolutionized remote sensing image classification by eliminating the need for handcrafted features^35^. CNNs can automatically learn hierarchical feature representations from raw data, leading to improved classification performance. Pre-trained CNNs, such as AlexNet, VGG, and ResNet, have been fine-tuned for remote sensing image classification tasks, demonstrating significant improvements in classification accuracy^28,36^. For example,^37^ introduces a novel deep learning-based method for forest change detection, which effectively distinguishes between changed and unchanged areas by enhancing the Forest Fused Difference Image (EFFDI) and applying the Recurrent Residual U-Net. Domain-specific CNN architectures, such as U-Net and SegNet, have also been proposed for remote sensing image classification, addressing unique challenges in this field, such as varying spatial resolutions and complex class structures^38,39^. Despite the numerous advantages of deep learning-based approaches, significant challenges remain in HRI classification. End-to-end semantic segmentation networks are predominantly employed in deep learning-based remote sensing image classification to accomplish pixel-level classification^40^. However, for complex feature objects, these semantic segmentation methods exhibit a “pretzel effect,” as accurately determining the appropriate class for each pixel can be quite difficult^21,22^.

Presently, numerous studies employ object-based segmentation combined with deep neural network approaches for HRI classification. Such methods circumvent the need for intricate, artificially designed features and enhance classification accuracy. While these object-based classification techniques can achieve higher accuracy through deep learning networks, determining the segmentation scale remains a challenge due to the network output size, potentially leading to over-segmentation or under-segmentation issues. Superpixel segmentation, which groups adjacent pixels into irregular pixel blocks with uniform distribution, has demonstrated effectiveness in HRI classification.^25^ introduced a deep learning approach that relies on CNNs and energy-driven sampling for HRI classification.^26^ employed a deep neural network technique for standardized segmentation of objects in HRI classification. These superpixel-centric methods can proficiently outline and represent the features of high spatial resolution images.

### Transformer

The Transformer model, proposed by^27^, has revolutionized the field of natural language processing and has been successfully applied to various tasks, such as machine translation, sentiment analysis, and named entity recognition.

In recent years, the integration of Transformer models with conventional approaches has marked a significant advancement in remote sensing image classification. The HyFormer framework, proposed by Yan et al.^41^, exemplifies this trend by merging Transformer models to bolster feature expressiveness for pixel-level multispectral image classification. Similarly, Xu et al.^42^introduced a novel network leveraging multiscale and cross-level attention learning (MCAL) for hyperspectral image (HSI) classification. This approach capitalizes on both global and local multiscale features through a multiscale feature extraction module coupled with a cross-level feature fusion module, enhancing the precision of HSI classification. Another innovative model, the SS-TMNet, developed by Huang et al.^43^, integrates spatial-spectral Transformer with multi-scale convolution. This network excels in extracting comprehensive local and global spatial-spectral information for HSI classification, showcasing the potential of spatial-spectral analysis in remote sensing. These developments underscore the transformative impact of Transformer-based models in remote sensing, offering novel methodologies for accurate and efficient image classification.

The core ideas of Transformer-based models revolve around self-attention mechanisms, positional encoding, and layer normalization, which effectively capture long-range dependencies in input data. This paper discusses the application of the Transformer architecture in conjunction with superpixel segmentation for remote sensing image classification, aiming to improve the performance of high-resolution satellite image (HRSI) classification.

The Transformer model is built upon the self-attention mechanism, which enables the model to weigh the importance of different input elements relative to each other. This mechanism is particularly useful for capturing long-range dependencies in data, as it enables the model to focus on relevant parts of the input sequence while disregarding less relevant parts. The self-attention mechanism is mathematically described as follows:

1 $$\begin{aligned} Attention(Q, K, V) = Softmax\left( \frac{QK^T}{\sqrt{d_k}}\right) V, \end{aligned}$$

where *Q*, *K*, and *V* are the query, key, and value matrices, respectively, and $d_k$ is the dimension of the key vector. The softmax function is applied to the dot product of the query and key matrices, normalized by the square root of $d_k$, which results in a probability distribution over the input elements. This distribution is then used to compute a weighted sum of the value vectors, generating the output of the attention mechanism.

In this work, we propose to integrate the Transformer model with superpixel segmentation for remote sensing image classification. The goal is to leverage the long-range dependency capturing capabilities of the Transformer model to improve the classification performance of HRI by combining the superpixel.

**Figure 2:**
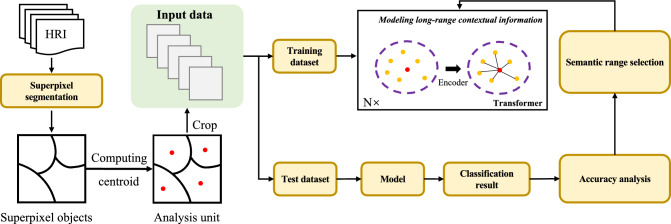
Flowchart for high-resolution remote sensing image classification using joint superpixel and Transformer.

**Figure 3:**
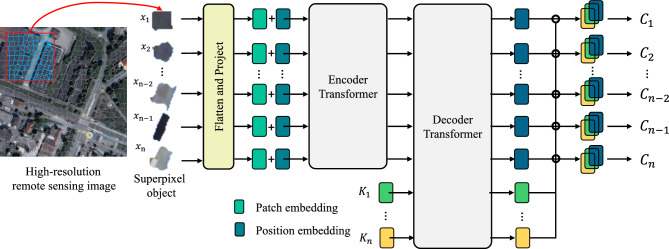
Graphical representation of combining superpixel and Transformer framework. JST has two components. 1. HRI are segmented into homogeneous objects by a superpixel segmentation algorithm. 2. Superpixel objects are linearly projected into tokens with position information. The added features are then processed by the encode-Transformer and decode-Transfomer modules, which have multiple self-attention layers and can extract contextual dependency information between objects and finally obtain the category of each object by SoftMax.

## Methods

### Overall framework

Figure 2 shows the technical flowchart of the joint superpixel segmentation and Transformer for HRI classification, which mainly includes: (1) superpixel segmentation. HRI are segmented by a simple linear iterative clustering segmentation algorithm to obtain superpixel objects. Superpixel objects are then used as the input of the network. (2) Model training and classification. The network framework is shown in Figure 3. The model is based on a proposed encoder- and decoder Transformer structure that maps a sequence of patch embeddings to pixel-level tokens for extracting features, and finally outputs the category of each input object. the proposed encoder- and decoder Transformer structure is described in detail in Section 2.3.

**Figure 4:**
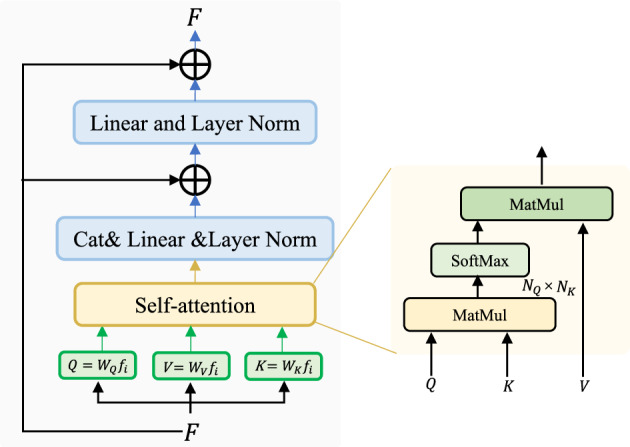
A layer in Transformer encoder and decoder.

### Superpixel segmentation

The simple linear iterative clustering (SLIC) algorithm is a superpixel segmentation method that considers color space and spatial distance. Firstly, the image’s color space is transformed into CIELab, and initial clustering centers are sampled at intervals of *S* pixels. The clustering points’ distance is set as $S=\sqrt{\frac{N}{k}}$ to produce superpixels of approximately the same size, where *k* is the desired number of superpixels, the *N* represents the total number of pixels in the image. The clustering centers are then moved to the lowest gradient in a $3 \times 3$ domain.

The algorithm involves initializing seed points (clustering centers) according to the set number of superpixels and distributing them evenly within the image. Next, seed points are reselected within an $n \times n$ neighborhood, and the gradient values of all pixel points in the neighborhood are computed. Seed points are then moved to the location with the smallest gradient in the neighborhood.

Each pixel point is assigned class labels within the neighborhood around each seed point, limiting the search range to $2S \times 2S$. The distance metric includes color and spatial distance, calculated for each searched pixel point as the distance to the seed point. The color distance $d_{c}$ is given by:

2 $$\begin{aligned} d_{c}=\sqrt{\left( l_{j}-l_{i}\right) ^{2}+\left( a_{j}-a_{i}\right) ^{2}+\left( b_{j}-b_{i}\right) ^{2}} \end{aligned}$$

The spatial distance $d_{s}$ is given by:

3 $$\begin{aligned} d_{s} =\sqrt{\left( x_{j}-x_{i}\right) ^{2}+\left( y_{j}-y_{i}\right) ^{2}} \end{aligned}$$

The final distance metric $D^{\prime }$ is given by:

4 $$\begin{aligned} D^{\prime }=\sqrt{\left( \frac{d_{c}}{m}\right) ^{2}+\left( \frac{d_{s}}{S}\right) ^{2}} \end{aligned}$$

Since each pixel point is searched by multiple seed points, each pixel point is given a distance from the surrounding seed points, and the seed point corresponding to the minimum value is taken as the clustering center of that pixel point.

### Transformer

#### Encoder-Transformer

The superpixel object is split into a one-dimensional vector, where $H \times W$ denotes the superpixel object length and width and C is the number of channels. Then, *x* is linearly projected to a patch embedding $\textbf{x}_0=\left[ \textbf{E} x_1, \ldots , \textbf{E} x_N\right] \in {\mathbb {R}}^{N \times D}$, where $\textbf{E} \in {\mathbb {R}}^{D \times \left( P^2 C\right) }$. To obtain the location information of the input object, the learnable position embedding ${\text {pos}}=\left[ {\text {pos}}_1, \ldots , {\text {pos}}_N\right] \in {\mathbb {R}}^{N \times D}$ are added to the patch sequence to obtain the resulting tagged input sequence $z_0=x_0+{\text {pos}}$. A Transformer encoder consisting of z0 input to the designed L-layer is used to obtain the features of the remote context. A Transformer layer is composed of a multi-headed attention block.

Figure 4 illustrates the one-layer Transformer network structure. Given the input tensors *F*, then the input tensors are linearly transformed as $W^{q}, W^{k}, W^{v}$ for obtaining $q^{i}$, $k^{i}$, and $v^{i}$, i.e.

5 $$\begin{aligned} \left\{ \begin{array}{l}q^{i}=W^{q} \cdot a^{i} \\ k^{i}=W^{k} \cdot a^{i}, \quad i \in \{1,2,3\} \\ v^{i}=W^{v} \cdot a^{i}\end{array}\right. \end{aligned}$$

Let the matrix $A=\left( a^{1}, a^{2}, a^{i}\right) , Q=\left( q^{1}, q^{2}, q^{i}\right) , K=\left( k^{1}, k^{2}, k^{i}\right) , V=\left( v^{1}, v^{2}, v^{i}\right)$, then *Q*, *K*, *V*

6 $$\begin{aligned} \left\{ \begin{array}{l}Q=W^{q} \cdot A \\ K=W^{k} \cdot A \\ V=W^{v} \cdot A\end{array}\right. \end{aligned}$$

Let the output matrix $B=\left( b^{1}, b^{2}, b^{i}\right)$, then *B*

7 $$\begin{aligned} B={\text {Attention}}(Q, K, V)=V \cdot {\text {softmax}}\left( \frac{K^{\top } \cdot Q}{\sqrt{d^{k}}}\right) \end{aligned}$$

The attention features obtained above are used to obtain the final output by the fully connected layer, batch normalization, and the input.

#### Decoder-Transformer

Our decoder transformer is inspired by ViT^44^, which introduces object embedding to generate instance masks. For Transformer decoder, we introduce a set of *K* learnable class embeddings $c = \left[ {\text {cls}}_1, \ldots , \textrm{cls}_K\right] \in {\mathbb {R}}^{K \times D}$, where *K* is the number of classes. Each class embedding is randomly initialized and assigned to a single semantic class. It will be used to generate class masks. The class embedding *c* is processed by the decoder jointly with the patch encoding. The decoder is a transformer encoder consisting of *M* layers. Decoder-Transformer generates the K-mask by computing the scalar product between the L2 normalized patch embedding $\textbf{z}_{\textbf{M}}^{\prime } \in {\mathbb {R}}^{N \times D}$ and the class embedding $\textbf{c} \in {\mathbb {R}}^{K \times D}$ output by the decoder. The class mask set is computed as follows:

8 $$\begin{aligned} {\text {Masks}}\left( \textbf{z}_{\textbf{M}}^{\prime }, \textbf{c}\right) =\textbf{z}_{\textbf{M}}^{\prime } \textbf{c}^T \end{aligned}$$

where ${\text {Masks}}\left( \textbf{z}_{\textbf{M}}^{\prime }, \textbf{c}\right) \in {\mathbb {R}}^{N \times K}$ are a set of patch sequences. *SoftMax* is applied on the class dimension, and layer norms are added to obtain pixel-level scores to form the final classification. The detailed structural parameters are described in Table 1.

**Table 1:** Parameters of each layer in a Vision Transformer (ViT) model.

Layer	Parameters	Description	Values/size
Input	Image size	Image dimensions	$H \times W \times C$
Patching	Patch size	Patch dimensions	$P \times P \times C$
Embedding	Embedding size	Embedding vector size	*D*
Transformer	Num. of layers	Number of transformer layers	*L*
	Num. of heads	Number of attention heads	$H_A$
	Hidden size	Hidden layer size	$D_H$
	Feed-forward	Feed-forward hidden size	$D_{FF}$
Classifier	Num. of classes	Number of output classes	$C_{Out}$

**Figure 5:**
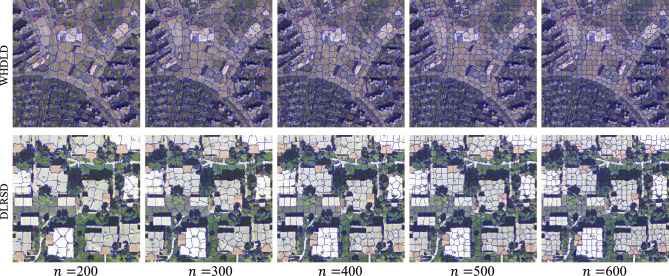
Superpixel segmentation results on partial WHDLD and DLRSD by different segmentation parameters, where *n* denotes the parameters used for the segmentation number of the superpixel objects.

## Data and parameter settings

### Data

*WHDLD*^45^ is a densely labeled dataset that can be used for remote sensing image retrieval and pixel-based tasks such as remote sensing classification. The images were meticulously extracted from satellite imagery provided by GaoFen-1 and ZiYuan-3 satellites. A key feature of this dataset is its spatial resolution, which stands at 2 meters. We use the pixels of each image with the following 5 category labels, namely buildings, roads, Bare ground and vegetation.

*DLRSD*^46^ is a densely labeled dataset of high-resolution remote sensing image classification dataset that can be used for semantic segmentation of remote sensing images. The images were sourced from the National Map and are primarily in the RGB colorspace. The spatial resolution for these images is set at 0.3 meter. We choose the image file (named mediumresidential) with 5 category labels, namely building, road, tree and vegetation in DLRSD for the experimental dataset.

### Parameter settings

Figure 5 shows the partial segmentation results on the two datasets with different segmentation parameters. The number of segmented superpixels is determined based on the size of the two images, and the tightness parameter is set to 1-60 for comparison. Figure 5 shows the segmentation results for the same tightness with different (*n*), where *n* denotes the number of superpixel objects. Since the influence of mixed pixels, increasing *n* can improve the homogeneity of segmented objects for WHDLD. The tightness of the image and the number of segmented objects in WHDLD are set to between 30-45 and 800 respectively, which is more suitable by comparison. For DLRSD, the tightness coverage parameter is set between 15-30 and the number of segmented objects is set to 650, which obtains better segmentation results. This is attributed to the clearer texture of the features covered in the DLRSD, reducing tightness and *n* can ensure the heterogeneity of each superpixel object.

The model is implemented by the Pytorch library and all the experiments are implemented on Ubuntu with 128GB RAM, RTX2070, 8GB. We indeed adopted an 80/10/10 split (Train/Validation/Test) for the datasets used in our study. The optimization model of the network is used with Adam and the learning rate, epoch and batch size were set to 0.0001, 4000 and 200.

## Experiment

### Evaluation matrix

Several widely used quantitative metrics, such as overall accuracy (OA), average accuracy (AA) and statistical Kappa coefficient ($\kappa$), are used to evaluate the performance of JST. OA represents the proportion of correctly classified test samples relative to all test samples, while $\kappa$ reflects the degree of agreement between the classification map generated by the considered model and the ground truth provided.

### Effect of the number of objects for input

Contextual dependencies exist between superpixel objects. How many input superpixel objects effect the classification accuracy is determined by the size of the range of contextual information that maximizes the identification of the categories between each object. The minimum number of input for segmented objects is 1 and the maximum number of input for segmented objects is the whole image. However, using one object as input does not provide more semantic information leading to misclassification. The number of input segmentation objects is chosen as 9, 12, 16, 20, 25, and 36 for evaluating the impact on classification accuracy. We use the same training parameters on both datasets from scratch to compare the impact of the input objects on the classification accuracy.

**Figure 6:**
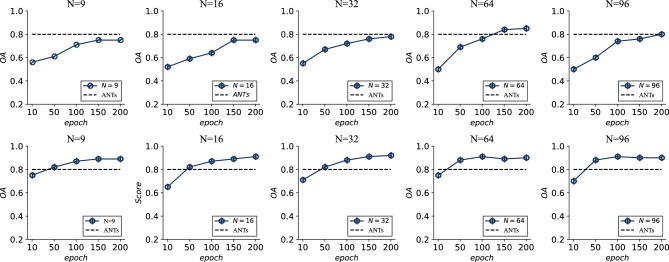
Overall accuracy for different number of input objects on WHDLD and DLRSD datasets. ANTs is the abbreviation of Accuracy Norm Threshold.

Figure 6 depicts the classification accuracies on the two datasets with a different number of inputs on the datasets. The results show the same trend of classification accuracy on both datasets, i.e., the overall trend of classification accuracy tends to increase as the number of inputs increases. When the number of input objects is increased from 96 to 128, there is little difference in the change of classification accuracy on WHDLD. For DLRSD, the overall decrease in classification accuracy on WHDLD is observed when the number of input objects is increased from 96 to 128. This may be because increasing the number of input objects increases the heterogeneity between objects in more distant neighborhoods and reduces the classification accuracy.

### Classification results

To demonstrate the performance of JST for HRI classification, we compare it with seven semantic segmentation models, namely UNet^47^, SegNet^48^, DeepLabV3+^49^, UPerNet^50^, SETR^51^, and Swin Transformer^52^. for a fair comparison, all models are trained on the same data set and trained from scratch.

**Table 2:** Overall accuracy (OA), average accuracy (AA), and Kappa coefficient ($\kappa$) achieved by different methods on WHDLD.

Method	Building	Road	Bare ground	Vegetation	Pavement	OA	AA	$\kappa$
UNet	$0.67 \pm 0.04$	$0.62\pm 0.02$	$0.65\pm 0.04$	$0.61\pm 0.09$	$0.56\pm 0.05$	0.65	0.65	0.64
SegNet	$0.71 \pm 0.06$	$0.66\pm 0.08$	$0.56\pm 0.07$	$0.61\pm 0.07$	$0.69\pm 0.05$	0.66	0.56	0.63
DeepLabV3+	$0.74 \pm 0.05$	$0.71\pm 0.07$	$0.68\pm 0.06$	$0.56\pm 0.07$	$0.75\pm 0.04$	0.71	0.60	0.70
UPerNet	$0.61 \pm 0.03$	$0.70\pm 0.01$	$0.61\pm 0.07$	$0.64\pm 0.01$	$0.63\pm 0.07$	0.65	0.59	0.61
SETR	$0.68 \pm 0.03$	$0.69\pm 0.06$	$0.54\pm 0.00$	$0.65\pm 0.01$	$0.62\pm 0.05$	0.69	0.61	0.68
Swin Transformer	$0.72 \pm 0.04$	${{0.79}}\pm { {0.02}}$	$0.76\pm 0.06$	$0.74\pm 0.05$	${{0.76}}\pm {{0.06}}$	0.76	0.62	0.70
JST	${{0.82}} \pm {{0.02}}$	$0.78\pm 0.03$	${{0.81}}\pm {{0.02}}$	${{0.80}}\pm {{0.01}}$	$0.75\pm 0.01$	${{0.79}}$	${{0.70}}$	${{0.78}}$

**Table 3:** Overall accuracy (OA), average accuracy (AA), and Kappa coefficient ($\kappa$) achieved by different methods on DLRSD.

Method	Building	Road	Tree	Vegetation	OA	AA	$\kappa$
UNet	$0.76 \pm 0.08$	$0.63\pm 0.04$	$0.73\pm 0.06$	$0.71\pm 0.02$	0.74	0.66	0.72
SegNet	$0.78 \pm 0.03$	$0.75\pm 0.02$	$0.74\pm 0.01$	$0.77\pm 0.09$	0.76	0.65	0.77
DeepLabV3+	$0.87 \pm 0.06$	$0.75\pm 0.04$	$0.73\pm 0.06$	$0.78\pm 0.06$	0.82	0.72	0.79
UPerNet	$0.84 \pm 0.07$	$0.74\pm 0.05$	$0.79\pm 0.04$	$0.82\pm 0.08$	0.79	0.70	0.78
SETR	$0.89 \pm 0.03$	$0.85\pm 0.01$	$0.84\pm 0.03$	${{0.87}}\pm {{0.05}}$	0.85	0.77	0.82
Swin Transformer	$0.88 \pm 0.04$	$0.82\pm 0.06$	$0.85\pm 0.02$	$0.80\pm 0.06$	0.86	0.80	0.85
JST	${{0.91}} \pm {{0.02}}$	${{0.90}}\pm {{0.01}}$	${{0.93}}\pm {{0.02}}$	$0.85\pm 0.05$	${{0.91}}$	${{0.85}}$	${{0.89}}$

Tables 2 and 3 report the accuracy and OA,$\kappa$ for each category using JST as well as other methods on the WHDLD and DLRSD datasets, respectively. it can be seen from the tables that JST performs the best on the WHDLD dataset with OA, AA and $\kappa$ of 0.79, 0.70 and 0.78, respectively. JST shows a significant improvement in performance over the other methods. The OA of JST increased by 17.72%, 16.45%, 10.12%, 17.72%, 12.65% and 3.80% compared to the other six compared models. UNet obtained a moderate performance with an overall classification of 0.65. The OA of SegNet, DeepLabV3+, UPerNet , and SETR is higher than that of UNet, however, overall OA is lower than JST. Swin Transformer rank second with an OA value of 0.76.

DLRSD covers features with higher contrast and clearer texture. Therefore, all methods have an overall improved classification performance, as shown in Table 3. JST provides the best performance on the DLRSD dataset with OA, AA, and $\kappa$ of 0.91, 0.85, and 0.89, followed by Swin Transformer. Since JST is built on superpixel segmentation, it retains the boundary information. Compared with other methods, JST not only obtains the context dependency but also preserves the boundary information of objects. In contrast, semantic segmentation algorithms rely entirely on the semantic information of each pixel in the dataset.

**Figure 7:**
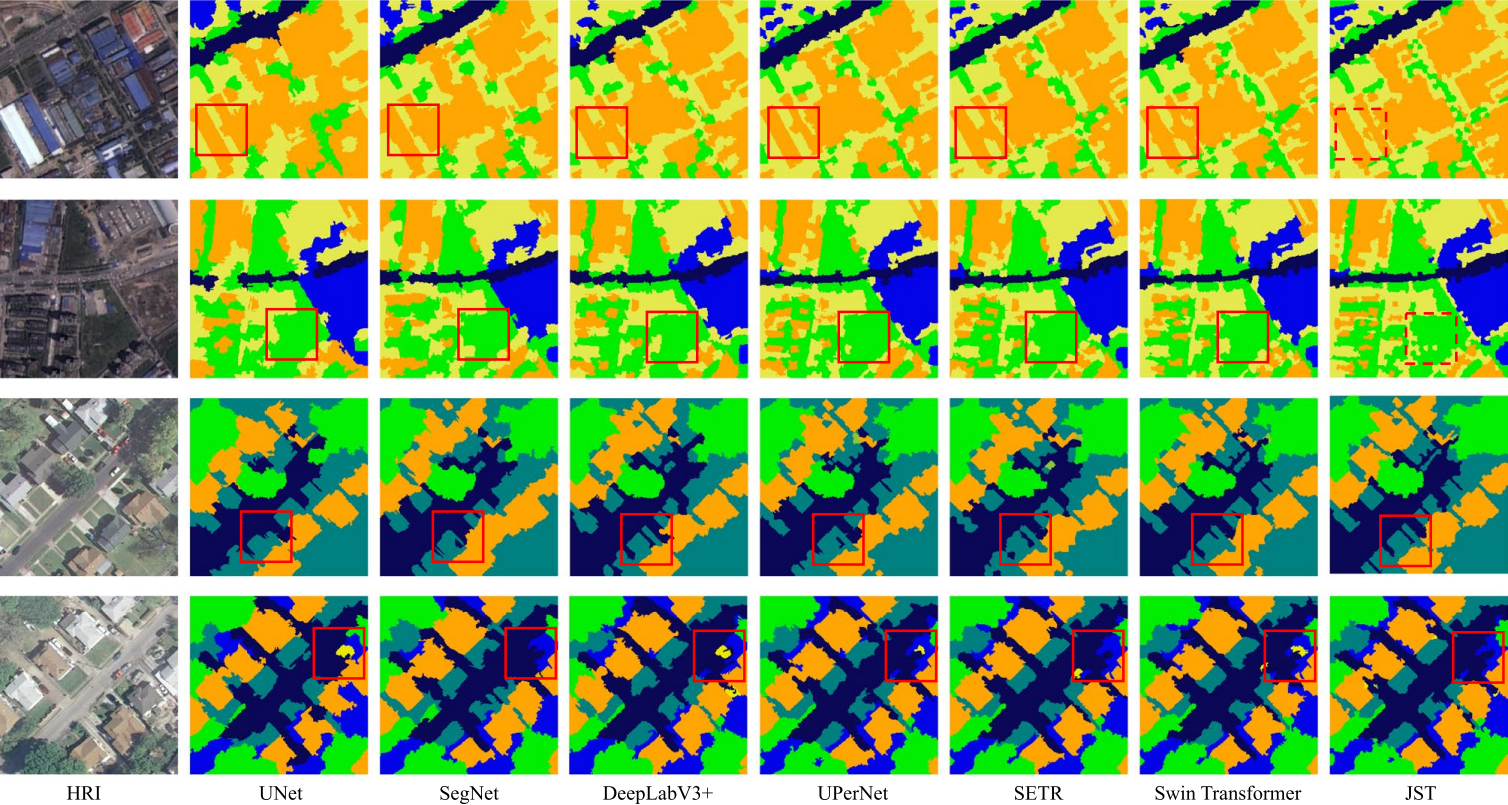
Qualitative classification results on WHDLD and DLRSD datasets. Areas are marked with red boxes for ease of inspection.

Figure 7 shows the classification results on WHDLD and DLRSD by JST and the comparison method. Visual inspection shows that JST outperforms the other six methods. Specifically, the first and second rows of Figure 7 show the classification results on the WHDLD. For the first row, the proposed method effectively delineates the classification boundaries on the highly similar building and ground categories. However, all comparison methods classify both ground and neighboring buildings into the same category, as shown in the red dashed box. In the second row, none of the comparison methods reflect the vegetation cover details and classify buildings as vegetation categories, as shown in the red dashed box. The classification performance of UNet on the WHDLD is overall lower than the other methods. Although Unet can roughly identify each category, misclassification is more serious. For example, there is a significant discontinuity in the road classification in the second row, which leads to a loss of detailed information. JST can correctly fit the boundary detail information of each category. Since we apply superpixel segmentation to extract homogenized objects, this preserves the boundary information of each category.

The third and fourth rows of Figure 7 show the classification results on DLRSD. Each category in the DLRSD dataset has rich texture information. Moreover, the contrast of each category is more obvious, which makes all methods have high classification accuracy. Similar to the WHDLD results, the proposed method can obtain more detailed classifications. As shown in the red dashed box in the third row, JST fits the boundaries of the road and vegetation completely. In addition, JST classifies the vehicles on the road completely into the road category, while the other methods fail. The vehicles are not classified in the training set. Therefore, it can be concluded that JST has a strong generalization capability.

### Ablation study

The number of input superpixel objects affects the classification accuracy as described in Section 4.1. In this section, we ablate different variants of our method on the WHDLD and DLRSD datasets. We investigate the effect of the number of model layers and the size of the tokens on classification accuracy. The network under each variant lets the random parameters (epoch time, learning rate, batch size) be deterministic during training. Evaluation of the overall classification accuracy on the test dataset is used to compare the effectiveness of the configured networks.

#### The number of layers of Transformer

We investigate the effect on the classification performance by varying the size of the layers and fixing the size of the tokens to 128. The detailed network combination and classification results are shown in Table 4. In fact, from layer number 9 to 11, we observe a 7.64% and 10.11% performance improvement for WHDLD and DLRSD, respectively. Finally, the classification model with the maximum number of layers achieves an OA of 0.80 and 0.87 on the WHDLD and DLRSD datasets. This trend suggests that increasing the number of layers is a strong source of improvement, however, this requires a balance between training efficiency and performance.

**Table 4:** Ablation study classification results on WHDLD and DLRSD, where *L*, *T* denotes the number of layers and token of the Transformer.

Data	Object	$T=128$				$L=12$		
		$L=9$	$L=10$	$L=11$	$L=12$	$T=192$	$T=256$	$T=384$
WHDLD	Building	$0.74\pm 0.03$	$0.76\pm 0.00$	$0.77\pm 0.01$	$0.81\pm 0.00$	$0.82\pm 0.04$	$0.82\pm 0.00$	$0.83\pm 0.00$
	Road	$0.71\pm 0.02$	$0.75\pm 0.01$	$0.77\pm 0.04$	$0.78\pm 0.03$	$0.79\pm 0.08$	$0.79\pm 0.02$	$0.78\pm 0.01$
	Bare ground	$0.73\pm 0.01$	$0.76\pm 0.02$	$0.78\pm 0.05$	$0.80\pm 0.02$	$0.80\pm 0.02$	$0.81\pm 0.01$	$0.79\pm 0.01$
	Vegetation	$0.72\pm 0.03$	$0.74\pm 0.01$	$0.76\pm 0.01$	$0.79\pm 0.01$	$0.79\pm 0.01$	$0.81\pm 0.03$	$0.82\pm 0.02$
	Pavement	$0.68\pm 0.02$	$0.70\pm 0.01$	$0.72\pm 0.01$	$0.75\pm 0.00$	$0.77\pm 0.06$	$0.77\pm 0.02$	$0.79\pm 0.06$
	OA	0.71	0.74	0.77	0.78	0.79	0.79	0.78
DLRSD	Building	$0.83\pm 0.02$	$0.85\pm 0.02$	$0.87\pm 0.03$	$0.88\pm 0.01$	$0.90\pm 0.01$	$0.90\pm 0.01$	$0.91\pm 0.01$
	Road	$0.80\pm 0.04$	$0.83\pm 0.01$	$0.85\pm 0.05$	$0.87\pm 0.00$	$0.89\pm 0.02$	$0.89\pm 0.02$	$0.90\pm 0.02$
	Tree	$0.83\pm 0.03$	$0.86\pm 0.02$	$0.89\pm 0.01$	$0.91\pm 0.04$	$0.92\pm 0.03$	$0.90\pm 0.01$	$0.91\pm 0.01$
	Vegetation	$0.78\pm 0.04$	$0.80\pm 0.03$	$0.83\pm 0.02$	$0.84\pm 0.03$	$0.84\pm 0.00$	$0.85\pm 0.02$	$0.86\pm 0.02$
	OA	0.81	0.83	0.86	0.87	0.91	0.91	0.90

#### Size of token

Since the input superpixel objects have different sizes, mapping these objects into random tokens, their sizes inevitably affect the classification accuracy. The average of the sizes of the superpixel objects we counted is 24 $\times$ 24. Therefore, we set the token sizes to 192, 256, 384 for comparing the classification performance on WHDLD and DLRSD respectively. When the token goes from 128 to 256, an improvement of 1.65% and 2.13% is obtained on the two datasets, respectively. For token 384, we also report classification results on WHDLD and DLRSD, which differ from token 256 by only 0.5% and 0.01%. These results show that increasing the size of the token does not significantly increase the classification accuracy, which is mainly dominated by the number of layers of Transformer that affect the classification.

## Conclusion

In this paper, we propose a joint superpixel segmentation and Transformer framework for HRI classification. The superpixel segmentation algorithm is used to obtain objects that are similar in size and homogeneous. A Transformer-based encoding and decoding structure is designed to obtain contextual dependencies between the input objects. The proposed method not only preserves the boundary information of superpixel segmentation, but also obtains a heterogeneous feature representation between objects. A comparison with six state-of-the-art methods is performed to show the superiority of the proposed method. In particular, tests are performed on the WHDLD and DLRSD datasets with OA, AA and $\kappa$ of 0.79, 0.70, 0.78 and 0.91, 0.85, 0.89, respectively. The proposed method provides an alternative solution for high-resolution remote sensing image classification.

JST involves a degree of manual intervention in the superpixel segmentation phase. However, this manual aspect may affect the overall efficiency of our method, particularly when considering the end-to-end classification process. Further research is warranted to explore ways to automate this process.

## Data Availability

The datasets generated and analysed during the current study are not publicly available due [The data are sourced from government classified projects] but are available from the corresponding author on reasonable request.

## References

[1] Zhong Y, Han X, Zhang L (2018). Multi-class geospatial object detection based on a position-sensitive balancing framework for high spatial resolution remote sensing imagery. ISPRS J. Photogramm. Remote. Sens..

[2] Huang B, Zhao B, Song Y (2018). Urban land-use mapping using a deep convolutional neural network with high spatial resolution multispectral remote sensing imagery. Remote Sens. Environ..

[3] Tong X-Y (2020). Land-cover classification with high-resolution remote sensing images using transferable deep models. Remote Sens. Environ..

[4] Zhu Q, Zhong Y, Zhang L, Li D (2018). Adaptive deep sparse semantic modeling framework for high spatial resolution image scene classification. IEEE Trans. Geosci. Remote Sens..

[5] Wen D (2021). Change detection from very-high-spatial-resolution optical remote sensing images: Methods, applications, and future directions. IEEE Geosci. Remote Sens. Mag..

[6] Zhu Q (2022). Land-use/land-cover change detection based on a Siamese global learning framework for high spatial resolution remote sensing imagery. ISPRS J. Photogramm. Remote. Sens..

[7] Zheng X, Chen T (2021). High spatial resolution remote sensing image segmentation based on the multiclassification model and the binary classification model. Neural Comput. Appl..

[8] Li Y, Zhang H, Xue X, Jiang Y, Shen Q (2018). Deep learning for remote sensing image classification: A survey. Wiley Interdiscip. Rev. Data Min. Knowl. Discov..

[9] Dong S, Wang P, Abbas K (2021). A survey on deep learning and its applications. Comput. Sci. Rev..

[10] Li L, Han L, Ding M, Cao H, Hu H (2021). A deep learning semantic template matching framework for remote sensing image registration. ISPRS J. Photogramm. Remote. Sens..

[11] Hosseiny B (2024). Beyond supervised learning in remote sensing: A systematic review of deep learning approaches. IEEE J. Sel. Top. Appl. Earth Obs. Remote Sens..

[12] Dai X (2021). Research on hyper-spectral remote sensing image classification by applying stacked de-noising auto-encoders neural network. Multimed. Tools Appl..

[13] Huang F, Yu Y, Feng T (2019). Hyperspectral remote sensing image change detection based on tensor and deep learning. J. Vis. Commun. Image Represent..

[14] Boulila W (2021). RS-DCNN: A novel distributed convolutional-neural-networks based-approach for big remote-sensing image classification. Comput. Electron. Agric..

[15] Zhao W (2017). Superpixel-based multiple local CNN for panchromatic and multispectral image classification. IEEE Trans. Geosci. Remote Sens..

[16] Gong M, Zhan T, Zhang P, Miao Q (2017). Superpixel-based difference representation learning for change detection in multispectral remote sensing images. IEEE Trans. Geosci. Remote Sens..

[17] Neupane B, Horanont T, Aryal J (2021). Deep learning-based semantic segmentation of urban features in satellite images: A review and meta-analysis. Remote Sens..

[18] Wu W, Li H, Li X, Guo H, Zhang L (2019). Polsar image semantic segmentation based on deep transfer learning-realizing smooth classification with small training sets. IEEE Geosci. Remote Sens. Lett..

[19] Li H (2022). Global and local contrastive self-supervised learning for semantic segmentation of HR remote sensing images. IEEE Trans. Geosci. Remote Sens..

[20] Li W, Chen H, Shi Z (2021). Semantic segmentation of remote sensing images with self-supervised multitask representation learning. IEEE J. Sel. Top. Appl. Earth Obs. Remote Sens..

[21] Yao J, Jin S (2022). Multi-category segmentation of Sentinel-2 images based on the Swin UNet method. Remote Sens..

[22] Li X, Liu B, Zhang K, Liu W (2021). Location soft-aggregation-based band weighting for hyperspectral image classification. IEEE Geosci. Remote Sens. Lett..

[23] Zhao Y, Yan F (2021). Hyperspectral image classification based on sparse superpixel graph. Remote Sens..

[24] Jia S (2019). Collaborative representation-based multiscale superpixel fusion for hyperspectral image classification. IEEE Trans. Geosci. Remote Sens..

[25] Lv X, Ming D, Chen Y, Wang M (2019). Very high resolution remote sensing image classification with seeds-CNN and scale effect analysis for superpixel CNN classification. Int. J. Remote Sens..

[26] Li L, Han L, Hu H, Liu Z, Cao H (2020). Standardized object-based dual CNNs for very high-resolution remote sensing image classification and standardization combination effect analysis. Int. J. Remote Sens..

[27] Vaswani A (2017). Attention is all you need. Adv. Neural inf. Process. Syst..

[28] Yan P, He F, Yang Y, Hu F (2020). Semi-supervised representation learning for remote sensing image classification based on generative adversarial networks. IEEE Access.

[29] Carranza-García M, García-Gutiérrez J, Riquelme JC (2019). A framework for evaluating land use and land cover classification using convolutional neural networks. Remote Sens..

[30] Lilay MY, Taye GD (2023). Semantic segmentation model for land cover classification from satellite images in Gambella National Park, Ethiopia. SN Appl. Sci..

[31] Prezelj J, Murovec J, Huemer-Kals S, Häsler K, Fischer P (2022). Identification of different manifestations of nonlinear stick-slip phenomena during creep groan braking noise by using the unsupervised learning algorithms k-means and self-organizing map. Mech. Syst. Signal Process..

[32] Zhang X, Han L, Han L, Zhu L (2020). How well do deep learning-based methods for land cover classification and object detection perform on high resolution remote sensing imagery?. Remote Sens..

[33] Peyghambari S, Zhang Y (2021). Hyperspectral remote sensing in lithological mapping, mineral exploration, and environmental geology: An updated review. J. Appl. Remote Sens..

[34] Wang J, Gao F, Dong J, Zhang S, Du Q (2022). Change detection from synthetic aperture radar images via graph-based knowledge supplement network. IEEE J. Sel. Top. Appl. Earth Obs. Remote Sens..

[35] He Z (2022). Hypervitgan: Semisupervised generative adversarial network with transformer for hyperspectral image classification. IEEE J. Sel. Top. Appl. Earth Obs. Remote Sens..

[36] Asokan, A. & Anitha, J. Machine learning based image processing techniques for satellite image analysis—a survey. In: *2019 International Conference on Machine Learning, Big Data, Cloud and Parallel Computing (COMITCon)*, 119–124 (IEEE, 2019).

[37] Khankeshizadeh E, Mohammadzadeh A, Moghimi A, Mohsenifar A (2022). FCD-R2U-net: Forest change detection in bi-temporal satellite images using the recurrent residual-based U-net. Earth Sci. Inf..

[38] Wang D (2022). A review of deep learning in multiscale agricultural sensing. Remote Sens..

[39] Jiang H (2022). A survey on deep learning-based change detection from high-resolution remote sensing images. Remote Sens..

[40] Haq MA, Rahaman G, Baral P, Ghosh A (2021). Deep learning based supervised image classification using UAV images for forest areas classification. J. Indian Soc. Remote Sens..

[41] Yan C (2023). Hyformer: Hybrid transformer and CNN for pixel-level multispectral image land cover classification. Int. J. Environ. Res. Public Health.

[42] Xu F, Zhang G, Song C, Wang H, Mei S (2023). Multiscale and cross-level attention learning for hyperspectral image classification. IEEE Trans. Geosci. Remote Sens..

[43] Huang X, Zhou Y, Yang X, Zhu X, Wang K (2023). SS-TMNet: Spatial-spectral transformer network with multi-scale convolution for hyperspectral image classification. Remote Sens..

[44] Dosovitskiy, A. *et al.* An image is worth 16x16 words: Transformers for image recognition at scale. arXiv preprint *arXiv:2010.11929* (2020).

[45] Shao Z, Zhou W, Deng X, Zhang M, Cheng Q (2020). Multilabel remote sensing image retrieval based on fully convolutional network. IEEE J. Sel. Top. Appl. Earth Obs. Remote Sens..

[46] Chaudhuri B, Demir B, Chaudhuri S, Bruzzone L (2017). Multilabel remote sensing image retrieval using a semisupervised graph-theoretic method. IEEE Trans. Geosci. Remote Sens..

[47] Ronneberger, O., Fischer, P. & Brox, T. U-net: Convolutional networks for biomedical image segmentation. In: *International Conference on Medical Image Computing and Computer-Assisted Intervention*, 234–241 (Springer, 2015).

[48] Badrinarayanan V, Kendall A, Cipolla R (2017). Segnet: A deep convolutional encoder-decoder architecture for image segmentation. IEEE Trans. Pattern Anal. Mach. Intell..

[49] Chen, L. -C., Zhu, Y., Papandreou, G., Schroff, F. & Adam, H. Encoder-decoder with atrous separable convolution for semantic image segmentation. In: *Proceedings of the European Conference on Computer Vision (ECCV)*, 801–818 (2018).

[50] Xiao, T., Liu, Y., Zhou, B., Jiang, Y. & Sun, J. Unified perceptual parsing for scene understanding. In: *Proceedings of the European Conference on Computer Vision (ECCV)*, 418–434 (2018).

[51] Zheng, S. *et al.* Rethinking semantic segmentation from a sequence-to-sequence perspective with transformers. In: *Proceedings of the IEEE/CVF Conference on Computer Vision and Pattern Recognition*, 6881–6890 (2021).

[52] Liu, Z. *et al.* Swin transformer: Hierarchical vision transformer using shifted windows. In: *Proceedings of the IEEE/CVF International Conference on Computer Vision*, 10012–10022 (2021).

